# Metabolomic and Microbial Remodeling by *Shanmei* Capsule Improves Hyperlipidemia in High Fat Food-Induced Mice

**DOI:** 10.3389/fcimb.2022.729940

**Published:** 2022-04-27

**Authors:** Lijing Du, Qian Wang, Shuai Ji, Yuanfang Sun, Wenjing Huang, Yiping Zhang, Shasha Li, Shikai Yan, Huizi Jin

**Affiliations:** ^1^ School of Pharmacy, Shanghai Jiao Tong University, Shanghai, China; ^2^ The Second Clinical College of Guangzhou University of Chinese Medicine, Guangzhou, China; ^3^ Institute of Traditional Chinese Medicine, Guangdong Pharmaceutical University, Guangzhou, China; ^4^ School of Traditional Chinese Medicine, Southern Medical University, Guangzhou, China

**Keywords:** *Shanmei* capsule, hyperlipidemia, metabolomics, intestinal microbial, regulation mechanism

## Abstract

Hyperlipidemia refers to a chronic disease caused by systemic metabolic disorder, and its pathophysiology is very complex. *Shanmei* capsule (SM) is a famous preparation with a long tradition of use for anti-hyperlipidemia treatment in China. However, the regulation mechanism of SM on hyperlipidemia has not been elucidated so far. In this study, a combination of UPLC-Q-TOF/MS techniques and 16S rDNA gene sequencing was performed to investigate the effects of *SM* treatment on plasma metabolism-mediated change and intestinal homeostasis. The results indicated that SM potently ameliorated high-fat diet-induced glucose and lipid metabolic disorders and reduced the histopathological injury. Pathway analysis indicated that alterations of differential metabolites were mainly involved in glycerophospholipid metabolism, linolenic acid metabolism, α-linoleic acid metabolism, and arachidonic acid metabolism. These changes were accompanied by a significant perturbation of intestinal microbiota characterized by marked increased microbial richness and changed microbiota composition. There were many genera illustrating strong correlations with hyperlipidemia-related markers (e.g., weight gains, GLU, and total cholesterol), including the *Lachnospiraceae* NK4A136 group and the *Lachnospiraceae* NK4B4 group. Overall, this study initially confirmed that hyperlipidemia is associated with metabolic disturbance and intestinal microbiota disorders, and SM can be employed to help decrease hyperlipidemia risk, including improving the abnormal metabolic profile and maintaining the gut microbial environment.

## Introduction

Hyperlipidemia is a chronic disease caused by systemic metabolic disorder where plasma lipid levels are abnormal, presenting the symptoms of an increase in low-density lipoprotein cholesterol (LDL-C), total cholesterol (TC), and triglyceride (TG) levels or a decrease in high-density lipoprotein cholesterol (HDL-C) (Centers for Disease Control and Prevention, 2011). Modern research indicates that hyperlipidemia is closely related to the progression of cardiovascular diseases, such as type 2 diabetes, hypertension, non-alcoholic fatty liver, and atherosclerosis (Nelson, 2013; Jung and Choi, 2014; Navar-Boggan et al., 2015; Okerson et al., 2017). At present, statins and fibrates are the most widely used lipid-lowering drugs for the treatment of hyperlipidemia, but their application has been limited due to high rates of several side effects and drug dependence (Choi and Shin, 2014; Mancini et al., 2016). As such, beneficial and secure therapeutic approaches are urgently required. Traditional Chinese medicine (TCM) has a long medicinal history with substantial clinical experience in lipid-lowering interventions, and it also harbors abundant resources for potential drug candidates. An increasing number of clinical studies suggest that TCM has an outstanding advantage in treating hyperlipidemia due to its stable clinic effect with low toxicity (Zhai et al., 2018; Wang et al., 2019). Different from Western medicine, TCM focused on multiple active ingredients and simultaneously targeted diverse physiological processes and pathways to promote the potentiality to maintain and restore health.

*Shanmei* capsule (SM) is a traditional Chinese patent medicine that is widely used to relieve numerous symptoms, including chest tightness or pain, palpitation, shortness of breath, and fatigue. It was approved by the Chinese Food and Drug Administration (Z20013183) for treatment of coronary heart disease, *Qi* stagnation, and blood stagnation syndrome in 2002 and listed in the Chinese Pharmacopoeia (Chinese Pharmacopoeia, 2020). SM is mainly composed of two medicinal herbs: crataegi folium (the leaves of *Crataegus pinnatifida* Bge. var. *major N.E. Br.* or *Crataegus pinnatifida* Bge.) and dahurian rose fruit (the fruit of *Rosa davurica Pall.* var. *davurica*) (Wang et al., 2021). Results from pharmacology research suggested that some component herbs of SM exhibited excellent hypolipidemic effects. Hyperoside is the major constituent and is listed by the Chinese Pharmacopoeia 2020 as a quality control indicator of SM. The crataegi folium extracts are rich in flavonoids, such as quercetin, hyperoside, and flavone glycosides, which are beneficial for decreasing blood lipid levels and glucose (Claudia et al., 2016; Dong et al., 2017). Due to its high vitamin content, dahurian rose fruit can be widely used in the production of foods, beverages, and beauty products. Apart from its nutritious value, dahurian rose fruit is also reported to have hypolipidemic and antioxidant effects (Jung et al., 2011; Huo et al., 2017). Clinically, SM has been found to reduce the risk of cardiovascular disease; however, little is known about the involving mechanism. Therefore, this study was designed to evaluate the underlying lipid-lowering mechanisms of SM in the treatment of hyperlipidemia.

There is growing evidence suggesting that perturbations of the intestinal microbiota and its influence on body metabolism and physiological functions may play a major role in various biological processes and disease development. For example, the intestinal microbiota contributes to the production of primary and secondary metabolites (e.g., bile acid biotransformation and short-chain fatty acid production) through processes including deconjugation, dehydroxylation, and fiber fermentation, and its dysregulation leads to a metabolic disturbance of the host system, especially those involving lipid and glucose metabolism (Thomas et al., 2009; Li et al., 2019). The community composition of intestinal microbiota is closely related to the metabolic characteristics of a variety of diseases, such as obesity, cardiovascular diseases, and inflammatory bowel disease (Aron-Wisnewsky and Clément, 2015; Weingarden and Vaughn, 2017; Sanna et al., 2019). Zhang et al. suggested that improving the intestinal microbiota is a promising strategy to ameliorating obesity (Zhang et al., 2019). As is well known, TCM directly or indirectly regulates intestinal microbiota composition and further plays a therapeutic effect. Therefore, investigation of the protective effects of SM against hyperlipidemia by regulating intestinal microbiota dysbiosis contributes to broadening the search for new and effective therapeutic drugs.

In this study, to verify the lipid-lowering effects of SM, high-fat diet (HFD)-induced hyperlipidemia mice were established and treated with SM, after which the plasma biochemical indicator changes were carefully observed. The metabolic signaling pathway and intestinal microbiota response of SM intervention were evaluated by an integrated approach combining metabolomics profiling and high-throughput 16S rDNA gene sequencing.

## Materials and Methods

### Materials

SM was obtained from Shanxi Taihang Pharmaceutical Co., Ltd. (Shanxi, China). Atorvastatin calcium salt trihydrate was supplied by Tokyo Chemical Industry Co., Ltd. (Tokyo, Japan). 12 hydration disodium hydrogen phosphate, potassium chloride, sodium chloride, potassium dihydrogen phosphate, and sodium dihydrogen phosphate were all purchased from Tianjin Zhiyuan Chemical Reagent Co., Ltd. (Tianjin China). The liver tissues of each mice were fixed in 4% paraformaldehyde at 4°C for 24 h, then the fixed tissues were embedded in paraffin and cut into 3-μm sections with a microtome (Leica, Nussloch, Germany). Paraformaldehyde (analytical grade) was supplied by Damao Chemical Reagent Factory (Tianjin). Hematoxylin–eosin (H&E) and neutral gum were purchased from Beijing Legend Biotech Co., Ltd. (Beijing, China) and Shanghai Yiyang Instrument Co., Ltd. (Shanghai, China), respectively. Triglyceride, total cholesterol, glucose, LDL-C, and HDL-C were measured by commercially available kits obtained from Shanghai Rongsheng Biotech Co., Ltd., while insulin was detected using kits supplied by Wuhan Huamei Biological Engineering Co. Ltd. (Wuhan, China).

### Animal and Sample Collection

A total of 60 mice C57BL/6 mice aged 3 weeks (Guangdong, China; Approval No.: SYXK-(Yue) 20120125) were commercially purchased from the Guangdong Medical Laboratory Animal Center. Animal protocols were reviewed and approved by the Laboratory Animals Ethics Committee of Guangdong Pharmaceutical University and conformed to the national animal ethics regulations (approval code: SPF2012264). All mice were raised under standard SPF conditions with *ad libitum* access to food and water. After 3 weeks of acclimatization, mice were randomly distributed into two groups, namely, control group (n = 15) and high-fat group (n = 45), which were fed with a standard chow diet and high-fat diet (60% of energy). High-fat diet (D12492) consisted of 60 kcal% fat, 20 kcal% protein, and 20 kcal% carbohydrate. A significant increase in plasma lipid level suggested that hyperlipidemia mice were established successfully after 12 weeks of high-fat diet feeding. Then, high-fat mice were randomly divided into three groups (with 15 mice in each group), including model group, *Shanmei* capsule (SM) group, and atorvastatin calcium (AT) group. Throughout the experiment, mice in the SM and AT groups were intragastrically administered a dosage of 0.0375 g/kg/day SM and 10 mg/kg/day AT one time every day, respectively. Meanwhile, the MD and control groups received the same volume of distilled water for 11 weeks. The body weight change per mice was logged once a week. At the end of the 11-week oral administration, the mice were fasted for 12 h, and then sacrificed to obtain plasma, fecal, and liver samples.

### Metabolomics Profiling of Plasma Samples

Prior to analysis, all of the plasma samples were thawed at room temperature and then centrifuged at 3,000 rpm at 4°C for 5 min to remove all sediment. Thereafter, 50 μl of the supernatant was transferred to a new EP tube and diluted with 200 μl acetonitrile (at a ratio of 1: 4), followed by vortexing for 1 min, and stored in a refrigerator at 4°C for 1 h. Finally, the diluted plasma samples were centrifuged at 4°C at 14,000 rpm for 15 min, and the supernatant was collected for further analysis.

Chromatographic analysis of plasma samples was performed on an ultra-performance liquid chromatography system (UPLC) (Agilent, Santa Clara, CA, USA) using an ACQUITY UPLC BEH C18 (2.1 mm × 100 mm, 1.7 μm). The column temperature was maintained at 30°C. The mobile phase was composed of 0.1% formic acid–water (v/v; A) and acetonitrile (B). The UPLC elution condition was set as follows: 0–5 min, 2%–42% B; 5–20 min, 42%–63% B; 20–25 min, 63%–86% B; 25–28 min, 86%–90% B; 28–36 min, 90%–98% B; 36–40 min, 98%–98% B. The flow rate was set at 0.4 ml/min, and the sample injection volume was 2 μl.

LC/MS analysis was performed on the Agilent 6545 quadrupole time-of-flight mass spectrometer coupled with an Agilent 1290 Liquid Chromatography II system. Data were acquired across a mass-to-charge (m/z) range of 50–2,000 m/z. The operation parameters were set as follows: drying gas temperature, 350°C; gas flow 7 l/min; nebulizer, 45 psi; capillary voltage, 3,000 V; cone voltage, 40 and 23 V; sheath gas temperature, 350°C; sheath gas flow, 11 l/min, collision energy, 10, 20, and 30 V; and scan rate, 3.0 spectra/s. All of the acquisition and analysis of data were monitored using Agilent MassHunter Acquisition software.

### Measurement of Plasma Biochemical Indicators and Hepatic Sections

After 11 weeks of treatment, oral glucose tolerance tests (OGTTs) were performed and the plasma was collected from mice which were overnight fasted (12 h). Then, the mice were orally administered 25% glucose in aqueous solution at 2 g/kg body weight. Blood glucose levels were measured sequentially using a GOLD-ACCU glucometer (Sinocare Inc., Changsha, China) at 0, 30, 60, and 120 min. Fasting insulin, TC, TG, LDL, and HDL were in accordance with the manufacturer’s protocols of corresponding commercial kits.

Freshly isolated mouse liver specimens were collected and fixed in 4% paraformaldehyde overnight and dehydrated by a dehydrator followed by paraffin embedding. Meanwhile, paraffin-embedded tissues were horizontally dissected into 4-mm tissue sections with a microtome, stained with hematoxylin–eosin (H&E), and then observed with a laboratory microscope (Carl Zeiss Inc., Germany). All specimen photographs were obtained using ×200 magnification to assess the histopathological changes.

### 16*s* rDNA Amplicon Sequencing

According to the manufacturer’s protocols, DNA libraries were quantified with a TruSeq Nano DNA LT Kit (Illumina, San Diego, CA, USA) and the quality of the library was assessed on an Agilent 2100 Bioanalyzer. Then, the V3–V4 regions of 16S rDNA were targeted amplified using the primers 341 F (5′-CCTACGGGNGGCWGCAG-3′) and 805 R (5′-GACTACHVGGGTATCTAATCC-3′) by PCR to obtain a microbial community structure and sequenced using an Illumina MiSeq platform (Illumina, San Diego, CA, USA) according to the 2 × 300-bp paired-end protocol. The amplicon sequencing data have been submitted to NCBI SRA (Submission ID, SUB9913815; BioProject ID, PRJNA741895).

### Statistical Analysis

Statistical analysis was performed using the Student’s t-test by GraphPad Prism 8 (GraphPad Software, La Jolla, CA, USA). The significance was set at 95% with a P-value of <0.05. Principal component analysis (PCA) and partial least square discriminant analysis (PLS-DA) were conducted using SIMCA-P software to cluster samples across groups. The LC-MS/MS data were acquired using Agilent Mass Hunter Workstation (*.d files) and processed in Agilent Profinder software (B.08.00) for retention time and mass alignment. The resulting features were then exported as *.cef files to Mass Profiler Professional 14.0 (Agilent Technologies, Santa Clara, CA, USA) for statistical analysis, including data alignment, filtering, and univariate, multivariate statistical, and differential analyses. Metabolites detected in at least ≥80% of one of two groups were selected for differential expression analyses. One-way ANOVA was performed to obtain differences between experimental groups. Relative metabolite quantitation was performed based on the peak area for each metabolite. Only the analytes with P values < 0.05 and fold change (FC) > 2 were treated as statistically significant.

The molecular formula that matched the criterion for ppm below 2 was considered as a potential candidate by the algorithm “Find Compounds by Formula” of Agilent MassHunter qualitative analysis software (B.07.01) and finally confirmed by examination of the MS/MS spectra. The pathway enrichment analysis was generated by MetaboAnalyst 5.0 (http://www.metaboanalyst.ca). Microbiome sequence analysis was performed using QIIME2. PICRUSt 2 analysis was designed to predict the functional composition profiles of gut bacteriome based on the MetaCyc database (https://metacyc.org). Correlations between microbiota and metabolites were analyzed by using the Spearman correlation method.

## Results

### SM Exhibits Robust Metabolic Protective Effects in HFD-Fed Mice

**Figure 1:**
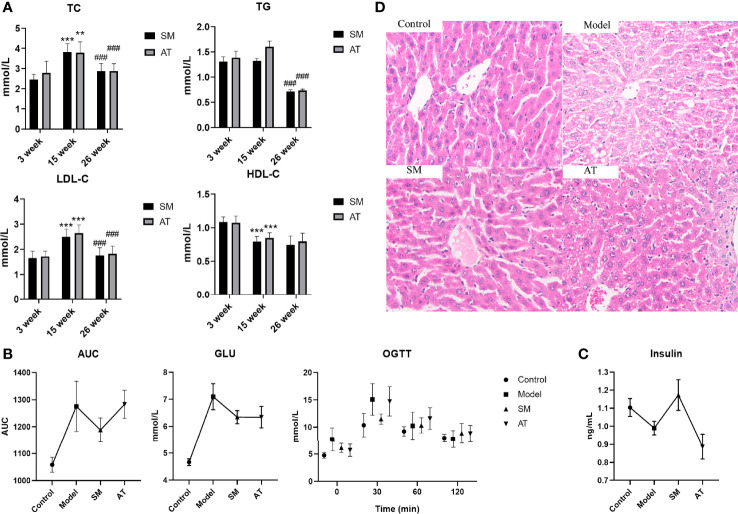
**(A)** Comparisons in TG, TC, LDL-C, and HDL-C between SM and AT groups. 3 weeks, 15 weeks, and 26 weeks represent before modeling, 12 weeks after modeling, and 11 weeks after drug administration, respectively. “*” represents a significant difference compared to 3 weeks and 15 weeks; “#” represents a significant difference compared to 15 weeks and 26 weeks. **(B)** Blood glucose levels and areas under the curve (AUC) during the oral glucose tolerance test (OGTT). **(C)** Change in insulin level. **(D)** Histopathologic sections of liver, H&E stain.

After the 11-week treatment, this study evaluated whether intragastric administration of *SM* exhibited a metabolic protective effect on HFD-fed mice. In terms of plasma lipid indexes, SM intake regulated HFD-induced abnormal lipid metabolism, with a significant decrease in the TG, TC, and LDL-C levels in SM-treated mice, whereas a large increase in HDL-C was not observed (**Figure 1A**). Moreover, SM not only restored the impaired glucose tolerance but also improved insulin. As expected, HFD-fed mice displayed impaired glucose tolerance and decreased insulin, as well as an evident increase in the fasting blood glucose and blood glucose at 0.5 h after meal as compared to control mice (**Figure 1B**). After 11 weeks of treatment, impaired glucose tolerance was ameliorated in the SM-treated compared to the model group, mainly manifested as a decrease in blood glucose level and the area under the curve (AUC) during OGTT (P < 0.05) (**Figure 1B**). Compared with the model group, the insulin level in the SM group was significantly increased (**Figure 1C**), suggesting increased insulin sensitivity. The pathology slice stained with H&E (**Figure 1D**) showed that the livers of MD mice exhibited cell swelling, obvious vacuoles, and mild hepatic steatosis. SM treatment could significantly reduce this phenomenon; the swelling degree was significantly reduced, and no obvious vacuoles were observed. Collectively, compared with the MD group, the pathological changes in liver tissues were significantly alleviated by SM and AT treatment. The metabolic protective effect of SM was the same as or even better than that of AT. Taken together, these findings exhibited that SM has a significant lipid-lowering effect in HFD-fed mice, reduced fat accumulation, decreased blood glucose level, improved insulin sensitivity, and protected liver function.

### Key Metabolic Pathways Involved in SM Treatment of HFD-Fed Mice

**Figure 2:**
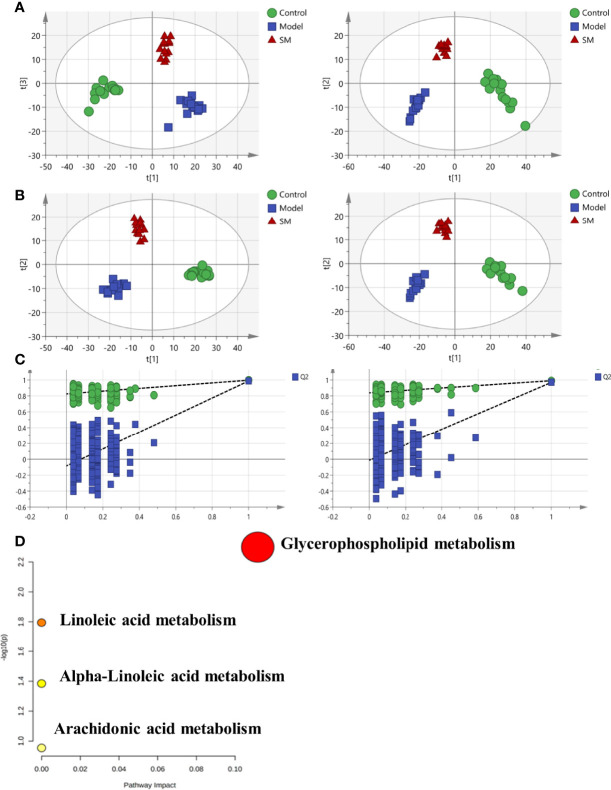
**(A)** Principal component analysis (PCA) model results in positive and negative modes. **(B)** Supervised analysis by PLS-DA. **(C)** Validation of the PLS-DA model by the 200-time permutation test. **(D)** KEGG pathway analysis for the identified metabolites.

To analyze the effects of SM treatment on metabolic pathways, a non-targeted plasma metabolomics study was conducted with UPLC-Q/TOF-MS. Multivariate statistics including unsupervised PCA and supervised PLS-DA were subsequently used to reveal clusters of different groups. Each dot in the PCA score plot is representative of an individual biological sample, and the results suggested that the three clusters were well separated (**Figure 2A**). A similar class-discriminating variation was again observed in the pattern recognition of PLS-DA (**Figure 2B**). The model classification parameters indicated the robustness and fitness of the established PLS-DA models (positive ion mode: R2Y = 0.995, Q2 = 0.971; negative ion mode: R2Y = 0.993, Q2 = 0.962). A permutation test (200 permutations) was further performed to prevent the overfitting on PLS-DA modeling. Results of the permutation test shown in **Figure 2C** indicated that no overfitting occurred in either the positive or negative mode.

According to the screening criteria (fold change ≥ 2, P < 0.05), a total of 13 differential metabolites were identified, including 9 upregulated and 4 downregulated. Results are presented in **Table 1**. The majority of identifiable metabolites consisted of lipids, including fatty acids, glycerolipids, glycerophospholipids, and sphingolipids. Notably, SM treatment abolished the effects of HFD-induced changes in metabolites. Pathway analysis indicated that alterations of these differential metabolites were mainly involved in glycerophospholipid metabolism, linolenic acid metabolism, α-linoleic acid metabolism, and arachidonic acid metabolism (**Figure 2D**).

**Table 1:** Identification of representative differential metabolites among different groups.

No.	Retention time (min)	Ion	Metabolites	Formula	Compound ID	Trend
1	6.257	Esi^+^	MG(15:0/0:0/0:0)	C_18_H_36_O_4_	HMDB11563	Down
2	6.482	Esi^+^	3,7-Dihydroxy-12-oxocholanoic acid	C_24_H_38_O_5_	HMDB00400	Up
3	8.464	Esi^+^	10,20-Dihydroxyeicosanoic acid	C_20_H_40_O_4_	HMDB31923	Up
4	12.444	Esi^-^	LysoPE[0:0/22:5(7Z,10Z,13Z,16Z,19Z)]	C_27_H_46_NO_7_P	HMDB11495	Up
5	12.516	Esi^+^	LysoPC[16:1(9Z)]	C_24_H_48_NO_7_P	HMDB10383	Up
6	15.616	Esi^-^	3-Ketostearic acid	C_18_H_34_O_3_	HMDB10736	Up
7	23.066	Esi^-^	Punicic acid	C_18_H_30_O_2_	HMDB30963	Up
8	25.666	Esi^-^	Tetracosahexaenoic acid	C_24_H_36_O_2_	HMDB02007	Up
9	26.476	Esi^+^	PC(18;4(6z, 9Z, 12Z, 15Z)/20;1(11z)	C_46_H_82_NO_8_P	HMDB08242	Up
10	27.279	Esi^-^	11(Z),14(Z)-Eicosadienoic acid	C_20_H_36_O_2_	HMDB05060	Down
11	30.482	Esi^+^	SM(d18:1/14:0)	C_37_H_75_N_2_O_6_P	HMDB12097	Down
12	33.861	Esi^+^	PC14:0/20:3(5Z,8Z,11Z)]	C_42_H_78_NO_8_P	HMDB07881	Up
13	39.188	Esi^+^	PG[16:0/16:1(9Z)]	C_38_H_73_O_10_P	HMDB10571	Down

### Gut Microbiota Diversity and Community Composition Changes After SM Administration

**Figure 3:**
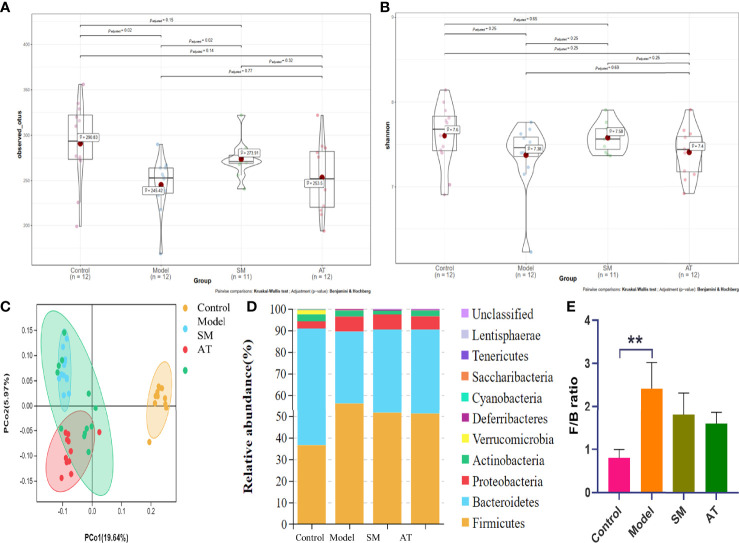
**(A)** The box plots of observed operational taxonomic unit (OTU) number. **(B)** Boxplot of Shannon diversity indices for the four groups. **(C)** Unweighted UniFrac PCoA results for all samples. **(D)** Stack bar charts of species distribution at the phylum level. **(E)** Ratio of Firmicutes to Bacteroidetes (F/B). **: significant difference (P < 0.01).

Increasing evidence suggests that intestinal microbiota may have a substantial effect on the HFD-induced model. To test it, 16S rDNA sequence analysis (hypervariable region V3–V4) was performed to evaluate the effect of SM on intestinal microbiota composition and abundance in fecal samples. As shown in **Figure 3A**, compared to mice in the model group, mice in the SM group and AT group presented an increase in the number of operational taxonomic units (OTUs). In addition, statistical analysis of the alpha diversity index (Shannon) indicated that diversity and richness exhibited an increasing trend in the SM and AT groups, compared to the model group (**Figure 3B**). The overall community structures of gut microbiota among the four groups were analyzed *via* unweighted UniFrac PCoA at the OTU level (**Figure 3C**). The separation in the scatterplot revealed the significant differences in gut microbiota composition between control, MD, and SM groups. After 11 weeks of HFD feeding, widespread changes in the community structures were attenuated at the phylum level (**Figure 3D**), with a significantly increased proportion of Firmicutes and a decreased abundance of Bacteroidetes. The ratio of Firmicutes to Bacteroidetes (Firmicutes/Bacteroidetes, F/B) was greatly increased in the MD group than in the control group (**Figure 3E**). SM treatment led to a perceivable reverse of such changes.

**Figure 4:**
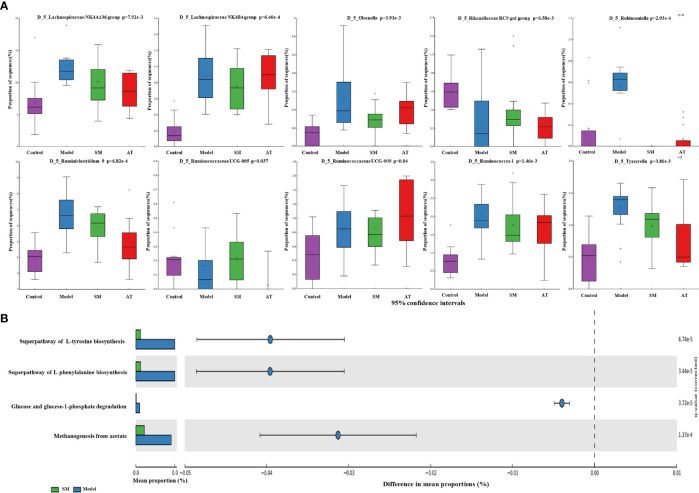
**(A)** Relative abundance of the predominant bacteria at the genus level. **(B)** Prediction of changed METACYC pathways using PICRUST 2 analysis. * In the Figure represents mean value.

To further identify the specific bacterial taxa at the genus level associated with hyperlipidemia, differences in intestinal microbiota between groups were explored using the Kruskal–Wallis (KW) rank-sum test. Based on the genus-OTU dataset, we discovered 10 significantly different genera (P < 0.05) (**Figure 4A**). Among them, the Lachnospiraceae NK4A136 group, Lachnospiraceae NK4B4 group, Olsenella, Robinsoniella, Ruminiclostridium 9, Ruminococcaceae UCG-009, Ruminococcus 1, and Tyzzerella were significantly more abundant in the fecal microbiota of the MD group compared the control group, while the Rikenellaceae RC9 gut group and Ruminococcaceae UCG-005 were significantly less abundant in the fecal microbiota of the MD group. SM treatment could markedly reverse these changes. These results indicated that SM modulates the intestinal microbiota of HFD-fed mice, resulting in the composition of microbiota similar to that of control group mice.

PICRUSt 2 analysis was conducted to analyze the METACYC pathways related to the intestinal microbiotas at the genus level. As shown in **Figure 4B**, the METACYC pathway analysis showed that amino acid metabolism and glucose metabolism were enriched in the SM group compared to the MD group. Amino acid biosynthesis superpathways, such as the superpathway of L-tyrosine biosynthesis and the superpathway of L-phenylalanine biosynthesis, were highly detected in the SM group. Glucose and glucose-1-phosphate degradation pathways were largely associated with glycogen and glucose metabolism.

### Correlation of Gut Microbiota and Metabolites

To evaluate potential interactions across the differentially abundant intestinal microbiotas at the genus level, a SparCC correlation network plot was constructed. Red and blue connecting lines correspond to positive and negative correlations, respectively. The complex network suggested a few specific intestinal microbial taxa that were closely related to other portions of the microbial community.

**Figure 5:**
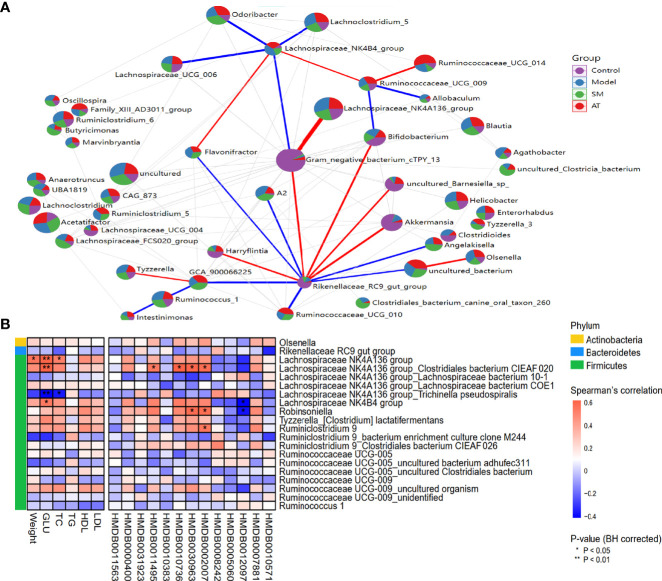
**(A)** Network between abundant sequences at the genus level built from SparCC correlation coefficients. **(B)** Heatmap of Spearman correlation among hyperlipidemia-related indexes, identified metabolites, and intestinal microbiota at genus level. For P-values of correlations, * indicated statistically significant correlation (*P < *0.05*; **P < 0.01).

A Spearman correlation heatmap was used to represent the relationship between perturbed intestinal microbiotas, biochemical indicators, and altered plasma metabolites, as presented in **Figure 5A**. At the phylum level, analyses showed that Firmicutes was highly correlated with hyperlipidemia. At the genus level, there were many genera illustrating strong correlations with hyperlipidemia-related markers (e.g., weight gains, GLU, and TC), including the Lachnospiraceae NK4A136 group and Lachnospiraceae NK4B4 group (**Figure 5B**). The Lachnospiraceae NK4A136 group, Robinsoniella, and Ruminiclostridium 9 exhibited an apparent positive correlation with altered plasma metabolites (e.g., LysoPE(0:0/22:5(7Z,10Z,13Z,16Z,19Z), 3-ketostearic acid, punicic acid, and tetracosahexaenoic acid), while the Lachnospiraceae NK4B4 group and Robinsoniella showed a robust negative relationship with SM(d18:1/14:0) (**Figure 5B**). In summary, these results indicated that the alterations of intestinal microbiotas induced by HFD were closely associated with hyperlipidemia-related indexes and altered the metabolomic profiles.

## Discussion

Hyperlipidemia refers to a chronic disease caused by systemic metabolic disorder, characterized by abnormal plasma lipid levels, and its pathophysiology is very complex. Feeding with HFD for 12 weeks caused a significant increase in plasma levels of TG, TC, and LDL-C, suggesting that the HFD-induced hyperlipidemia mouse model was successfully established. Results showed that SM treatment had a significant effect on reduction in fat accumulation and blood glucose level, increase in sensitivity and protect in liver function. We speculated that SM plays a hypolipidemic role *via* two different mechanisms. One potential mechanism is by rectifying the metabolic disturbance and maintaining the dynamic balance of metabolites. The other is *via* regulating intestinal microbiota and restoring intestinal homeostasis.

SM is mainly composed of two medicinal herbs: crataegi folium and dahurian rose fruit. Crataegi folium contains a variety of bioactive constituents that have a positive effect on humans, of which flavonoids (hyperoside, rutin, quercitrin, vitexin) are the most abundant (Wu et al., 2014; Wu et al., 2020). The reports from recent years have demonstrated that crataegi folium flavones possess antioxidative, antihypertensive, hypoglycemic, and hypolipidemic properties and are widely used in the clinic for the treatment of cardiovascular diseases, such as hyperlipidemia, hypertension, and coronary heart disease (Pang et al., 2021). The flavonoid fraction in crataegi folium could significantly inhibit the accumulation of TG and free fatty acids in 3T3-L1 preadipocytes by inhibiting the gene expression of C/EBPR, PPARγ, srebp1c, P2, and adiponectin (Li et al., 2015), which played an important role in the process of lipid metabolism. *Rosa davurica* pall, a member of the Rosaceae family, contains a large number of active components, including vitamins, flavonoids, tannins, and polyphenols, and has significant antioxidant, anti-inflammatory, and hypoglycemic activities. Shen et al. isolated the flavonoid-rich extracts from *Rosa davurica* Pall and investigated the anti-obesity activities. Results indicated that flavonoid-rich extract intervention significantly inhibited the body weight, liver weight, kidney weight, and epididymal adipose tissue weight of HFD-fed mice and lowered the plasma lipid levels (Shen et al., 2021). The results of our study are mostly consistent with previous research. In this study, the majority of identifiable metabolites consisted of lipids, including fatty acids, glycerolipids, glycerophospholipids, and sphingolipids. SM treatment abolished the effects of HFD-induced changes in lipid metabolites.

In this study, the differential metabolites in three groups (Control vs. Model vs. SM) were significantly enriched in pathways related to lipid metabolism, including glycerophospholipid metabolism, linolenic acid metabolism, α-linoleic acid metabolism, and arachidonic acid metabolism. Phospholipids are the most abundant lipids in the cell membrane with the fundamental structure features of phosphatidic acid and substituents. According to different substituents, it can be divided into phosphatidylcholine (PC), phosphatidylglycerol (PG), phosphatidylethanolamine (PE), and so on (Dalebroux, 2017). Lysophosphatidylcholines are the core metabolic intermediates of glycerophospholipid metabolism, which are deemed as independent risk factors in the progression of cardiovascular diseases (Paapstel et al., 2018). Notably, the elevated levels of LysoPE[0:0/22:5(7Z,10Z,13Z,16Z,19Z)], LysoPC[16:1(9Z)], PC[18;4(6z, 9Z, 12Z, 15Z)/20;1(11z), and PC(14:0/20:3(5Z,8Z,11Z)] observed in the plasma of SM-treated mice were remarkably decreased, suggesting that lipid accumulation in the hyperlipidemia animals was ameliorated. It is also found that the sphingomyelin SM (d18:0/14:0) level was increased in the SM group compared to that in the model group. Sphingolipids are essential structural constituents of intracellular lipids and involved in cell signaling and apoptosis (Merrill, 2011). Sphingomyelin accumulation has been related to type 2 diabetes and observed in mouse models of insulin resistance (Samad et al., 2006) as well as in the skeletal muscle of obese, insulin-resistant humans (Straczkowski et al., 2004).

We also found that N-3 polyunsaturated fatty acids were higher in SM-treated mice. The mechanisms by which N-3 polyunsaturated fatty acids regulate biological processes are commonly believed to begin with alpha-linolenic acid (18:3n-3) and end with the production of docosahexaenoic acid (DHA) (Sprecher, 2000). Tetracosahexaenoic acid (THA) is thought to be an important intermediate to docosahexaenoic acid (DHA) synthesis (Metherel et al., 2017). Naohiro Gotoh et al. showed that tetracosahexaenoic acid had the highest activity in inhibiting the accumulation of hepatic TG and reducing the increase in liver weight, and the order was higher than that of docosahexaenoic acid, docosapentaenoic acid, and eicosapentaenoic acid (Gotoh et al., 2018). Punicic acid, a polyunsaturated fatty acid also known as omega-5 (ω-5), is a strong inhibitor of tumor necrosis factor-alpha (TNFα)-induced priming of reactive oxygen species production and myeloperoxidase release by neutrophils (Tarek et al., 2009). It has been suggested that the punicic acid hypoglycemic effects are mediated by PPARγ, which might inactivate the pro-inflammatory pathway mediated by nuclear factor-kappa-B (NF-κB) and TNF-α (Hontecillas et al., 2009). Arachidonic acid is an essential polyunsaturated fatty acid and is the precursor for the synthesis of a variety of bioactive compounds (eicosanoids), such as leukotrienes, prostaglandins, and thromboxanes. These arachidonic acid-derived mediators have a very important role in inflammatory response, lipid metabolism, and functioning of the cardiovascular system (Hanna and Hafez, 2018; Sonnweber et al., 2018). Moreover, the regulation of bile acid was noted in mice treated with SM. 3,7-Dihydroxy-12-oxocholanoic acid is a kind of dihydroxy bile acid. Increased animal fat intake enhances the levels of secreted bile acids and cholesterol in bile. Bile acids are primarily responsible for the absorption of dietary fats and fat-soluble vitamins, as well as dissolution of cholesterol (Hofmann and Hagey, 2008). Our findings were in agreement with previous reports. These results suggest that SM treatment alleviated the plasma metabolite aberrations induced by hyperlipidemia and validated the role of SM in the modulation of lipid metabolic process.

There is substantial evidence that significant structural alterations of intestinal microbiota were developed in the pathogenesis of hyperlipidemia. Our findings showed that SM treatment improved the intestinal microbial richness and changed the microbiota composition. Widespread changes in the community structures were attenuated at the phylum level (**Figure 3D**), with a significantly increased proportion of Firmicutes and decreased abundance of Bacteroidetes. The ratio of F/B was highly correlated with energy metabolism. It is commonly accepted that the high ratio of F/B leads to higher energy absorption and further promotes the development of obesity (Koliada et al., 2017). At the genus level, the HFD-induced model group decreased the abundance of the Rikenellaceae_RC9_gut_group, Ruminococcaceae UCG-005, and Tyzzerella, each of which was associated with the risk of obesity and cardiovascular diseases. The Rikenellaceae_RC9_gut_group belongs to the dominant genera of Bacteroidetes, which is strongly associated with glucose metabolism parameters (Gao et al., 2020). SM treatment promoted the growth of beneficial bacterial genus Ruminococcaceae UCG-005, which was reported as a key genus for protecting against cardiovascular diseases (Li et al., 2021). Kelly et al. (Kelly et al., 2016) found that the abundance of Tyzzerella may be a risk factor to cardiovascular disease progression, indicating that the regulation of Tyzzerella by SM might improve health (Xu and Yang, 2020).

Correlations were also found in the study between intestinal microbiota and hyperlipidemia-related indexes. Several notable correlations were identified, including a positive correlation between plasma levels of LysoPE(0:0/22:5(7Z,10Z,13Z,16Z,19Z), 3-ketostearic acid, punicic acid, and tetracosahexaenoic acid with the Lachnospiraceae NK4A136 group, Robinsoniella, and Ruminiclostridium 9 and a negative correlation between Lachnospiraceae NK4B4 group and Robinsoniella with SM(d18:1/14:0). Several studies have linked these microbiota to obesity and increased cardiovascular risk (Holm et al., 2016; Liu et al., 2020; Wang et al., 2020). These data added to the evidence that intestinal microbiota may play an important role in hyperlipidemia risk. In light of this, the study initially confirmed that hyperlipidemia was associated with metabolic disturbance and intestinal microbiota disorders, and SM could be employed to help decrease hyperlipidemia risk, including improving the abnormal metabolic profile and maintaining the gut microbial environment.

## Conclusion

This study is the first to investigate the intervening effects of SM on mice with HFD-induced hyperlipidemia using untargeted metabolomics combined with 16S rDNA gene sequencing analysis. Pharmacodynamic results demonstrated that the mouse model of hyperlipidemia was successfully established by HFD, and the metabolic protective effect could be improved by SM treatment, including reducing fat accumulation, decreasing blood glucose level, improving insulin sensitivity, and protecting liver function. Moreover, we also compared the effect of SM with a positive control statin. Over the 11 weeks of treatment, the SM group exhibited a similar or even greater protective effect than the AT group did. The involved lipid-lowering effect may consist of the following two aspects. On the one hand, SM rectified the metabolic disturbance to maintain the dynamic balance of metabolites, mainly involved in glycerophospholipid metabolism, linolenic acid metabolism, α-linoleic acid metabolism, arachidonic acid metabolism. On the other hand, the beneficial effects of SM are mostly attributed to modulation of the intestinal microbiota, which is pivotal in multiple indexes related to hyperlipidemia.

## Data Availability Statement

The original contributions presented in the study are publicly available in NCBI using accession number PRJNA741895.

## Ethics Statement

Animal protocols were reviewed and approved by the Laboratory Animals Ethics Committee of Guangdong Pharmaceutical University and conformed to the National animal ethics regulations (approval code: SPF2012264).

## Author Contributions

SY, HJ: supervising and coordinating the animal experiment and checking the manuscript. LD: analyzing the data and preparing the manuscript, checking language. QW, YS, YZ: performing the animal experiment. SJ: analyzing the data. SL, WH: evaluating the data and amending the manuscript. All authors contributed to the article and approved the submitted version.

## Conflict of Interest

The authors declare that the research was conducted in the absence of any commercial or financial relationships that could be construed as a potential conflict of interest.

## Publisher’s Note

All claims expressed in this article are solely those of the authors and do not necessarily represent those of their affiliated organizations, or those of the publisher, the editors and the reviewers. Any product that may be evaluated in this article, or claim that may be made by its manufacturer, is not guaranteed or endorsed by the publisher.

## Abbreviations

SM, Shanmei capsule; LDL-C, low-density lipoprotein cholesterol; TC, total cholesterol; TG, triglyceride; HDL-C, high-density lipoprotein cholesterol; TCM, traditional Chinese medicine; HFD, high-fat diet; AT, atorvastatin calcium; OGTTs, oral glucose tolerance tests; H&E, hematoxylin–eosin; PCA, principal component analysis; PLS-DA, partial least square discriminant analysis; AUC, areas under the curve.
