# Supplementary material for: Metabolomic and Microbial Remodeling by Shanmei Capsule Improves Hyperlipidemia in High Fat Food-Induced Mice
Source: Front Cell Infect Microbiol. 2022 Apr 27;12:729940. doi: 10.3389/fcimb.2022.729940 (PMC9094705; doi:10.3389/fcimb.2022.729940)

MS/MS fragmentation spectra of metabolites

(1) MG(15:0/0:0/0:0)

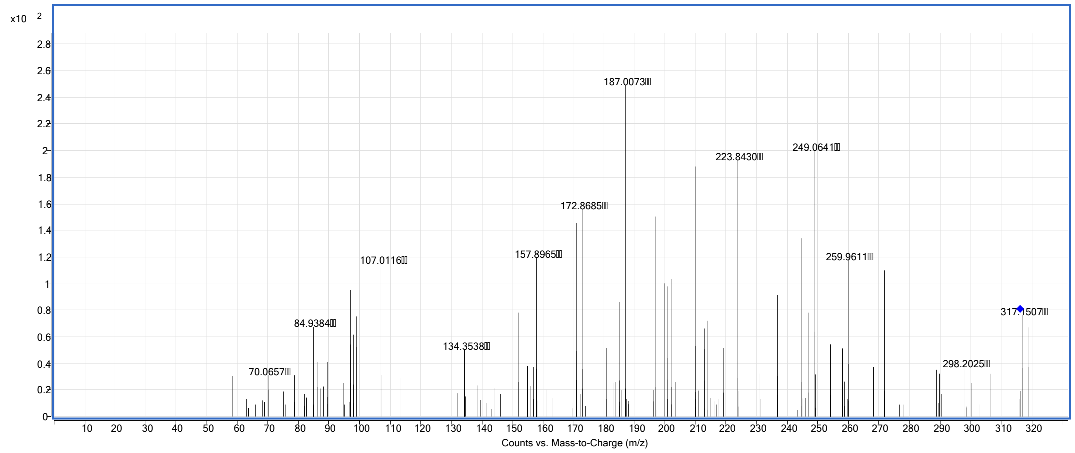

(2) 3,7-Dihydroxy-12-oxocholanoic acid

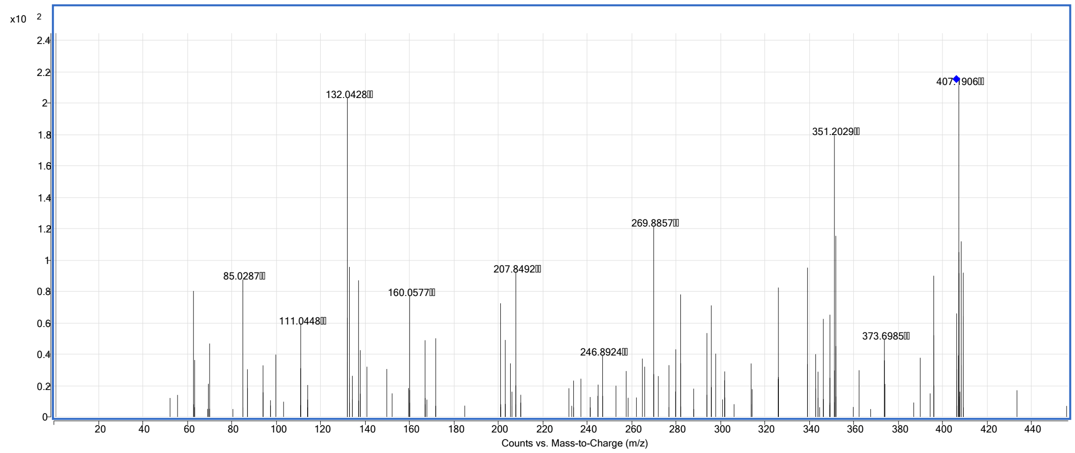

(3) 10,20-Dihydroxyeicosanoic acid

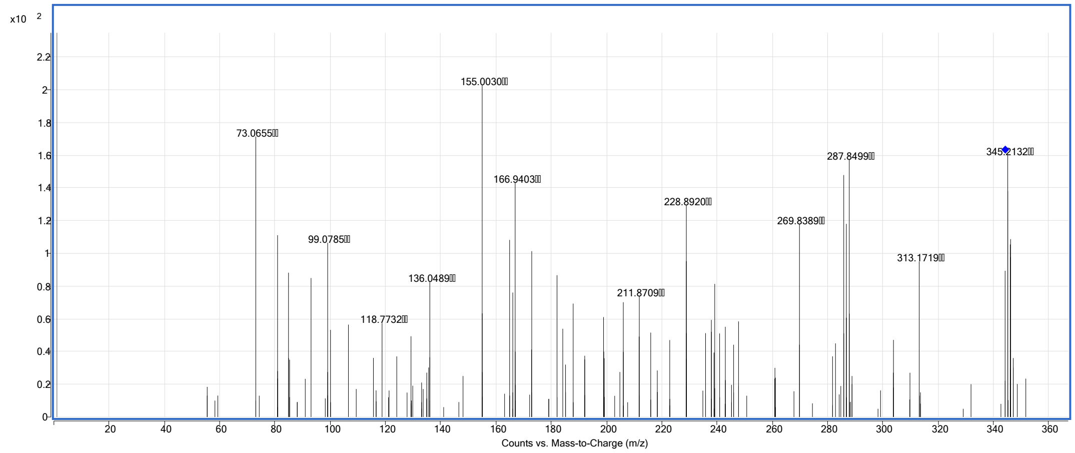

(4) LysoPE(0:0/22:5(7Z,10Z,13Z,16Z,19Z))

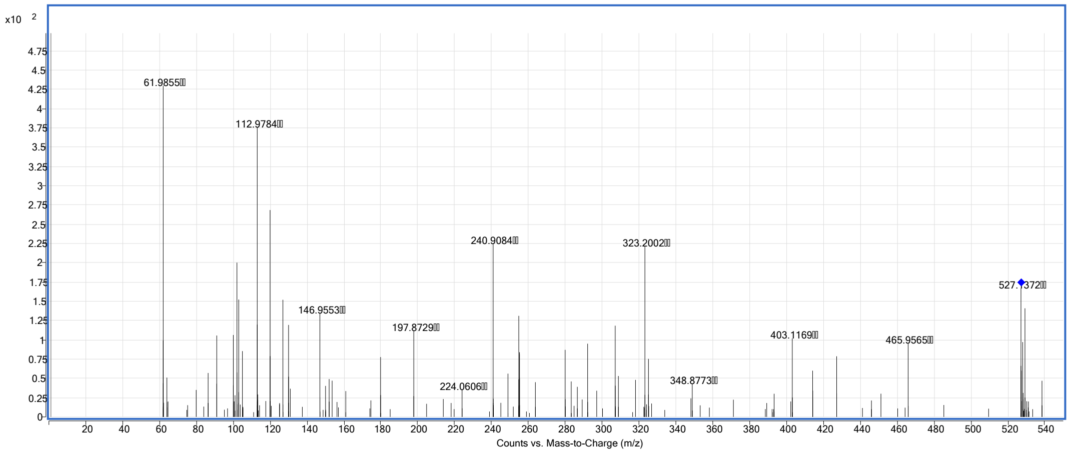

(5) LysoPC(16:1(9Z))

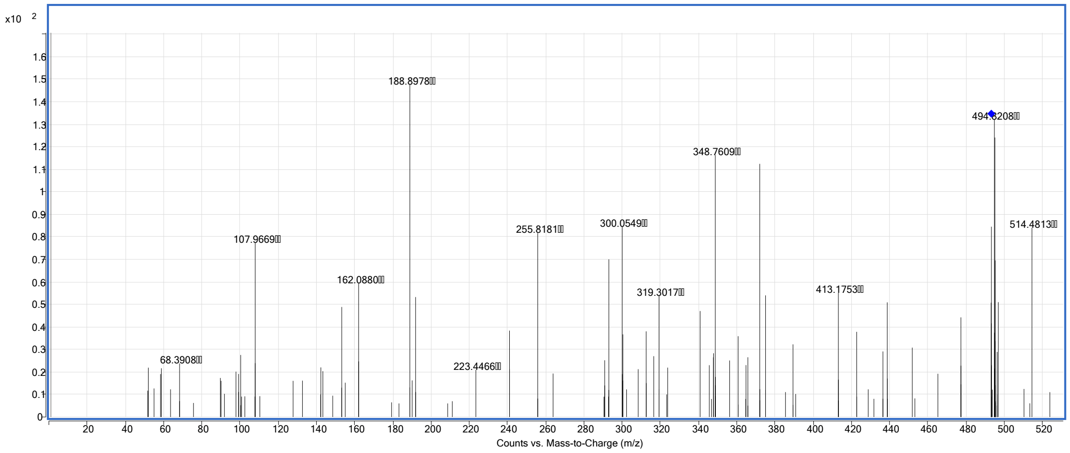

(6) 3-ketostearic acid

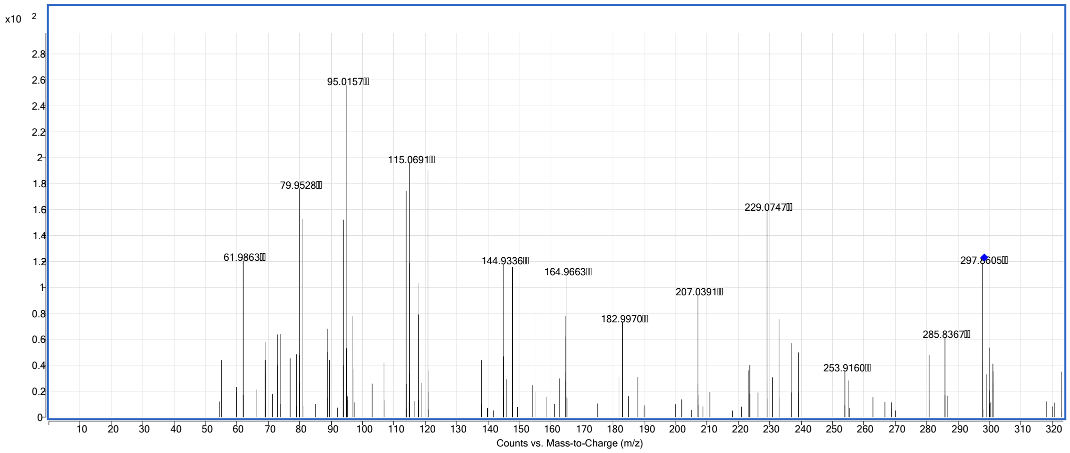

(7) Punicic acid

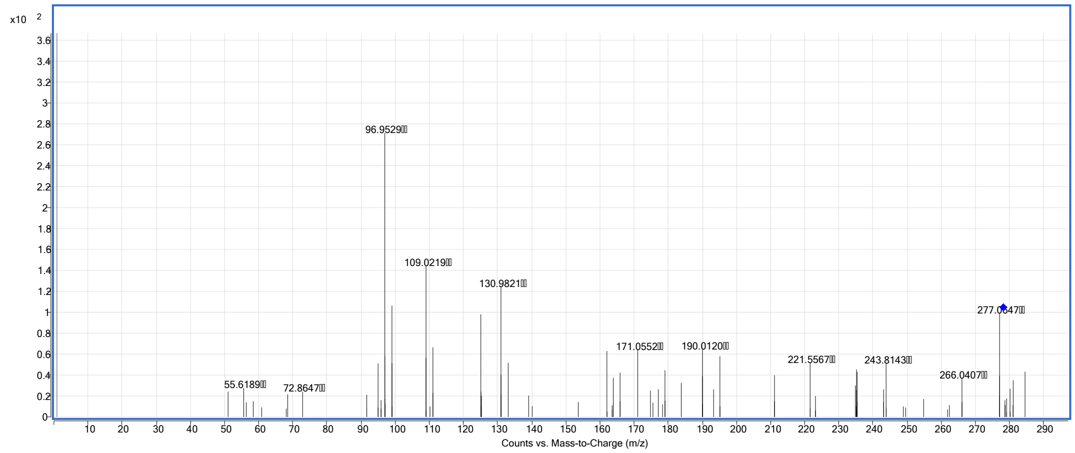

(8) Tetracosahexaenoic acid

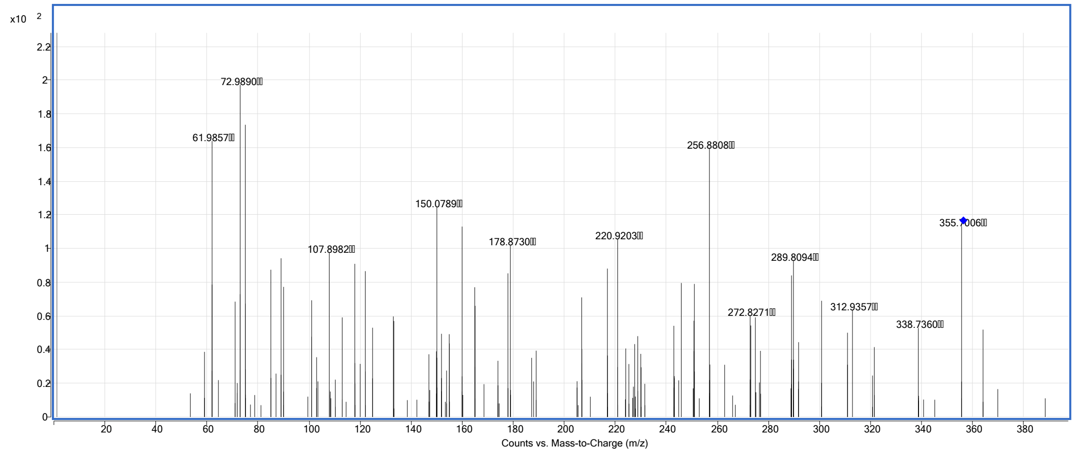

(9) PC(18：4(6z，9Z，12Z，15Z)/20：1(11z)

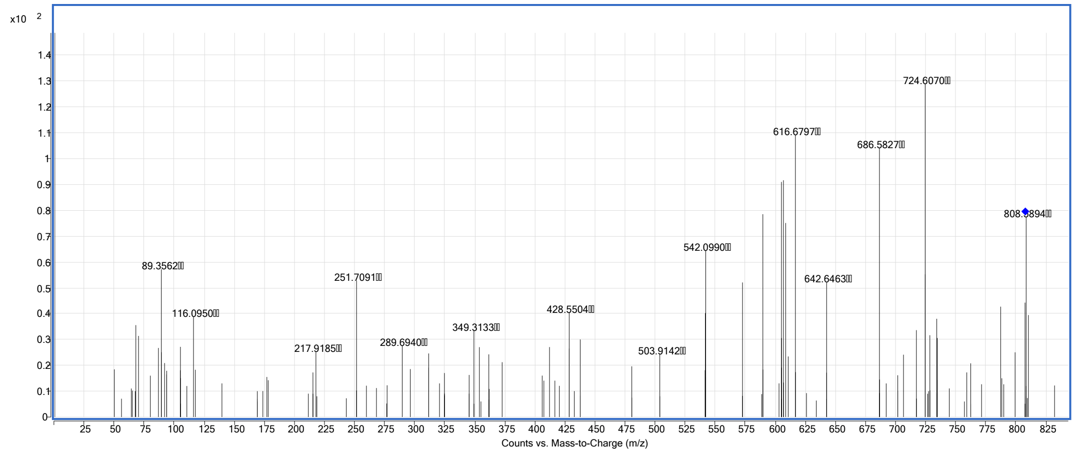

(10) 11(Z),14(Z)-Eicosadienoic Acid

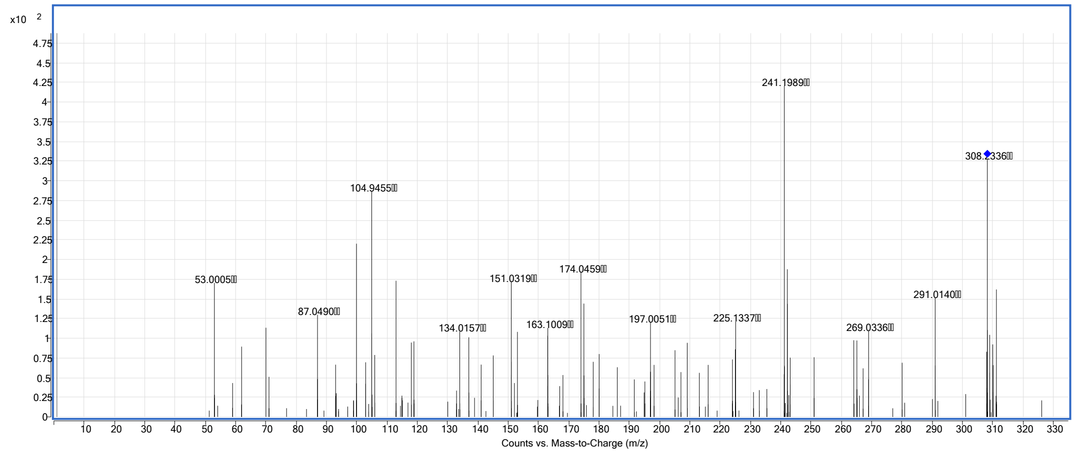

(11) SM(d18:1/14:0)

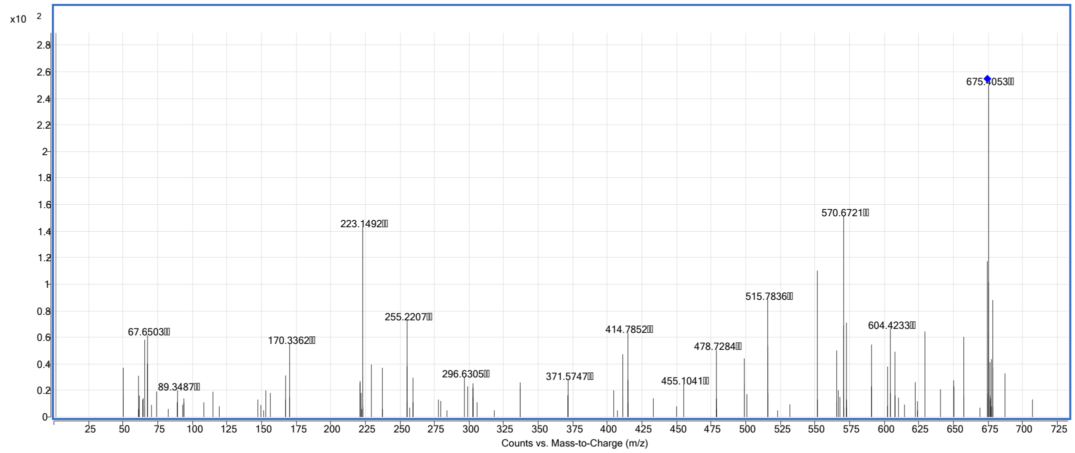

(12) PC(14:0/20:3(5Z,8Z,11Z))

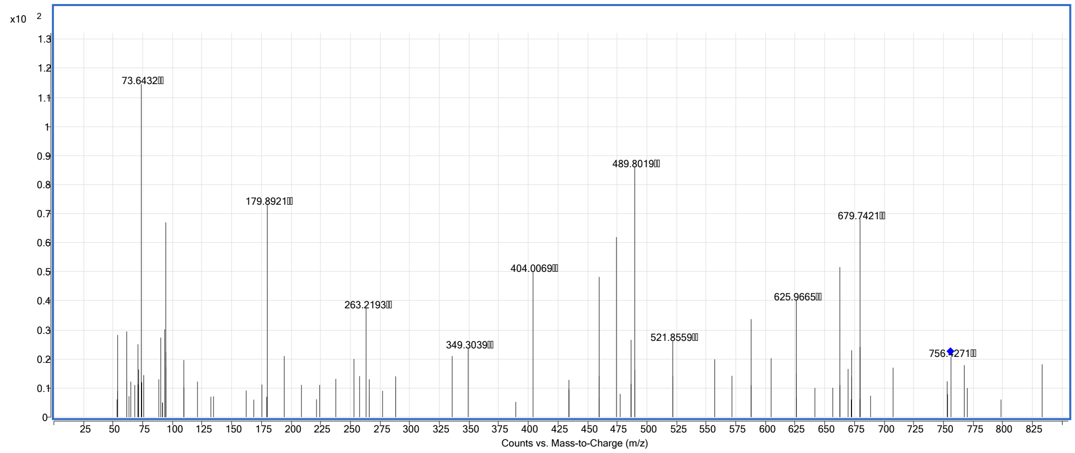

(13) PG(16:0/16:1(9Z))

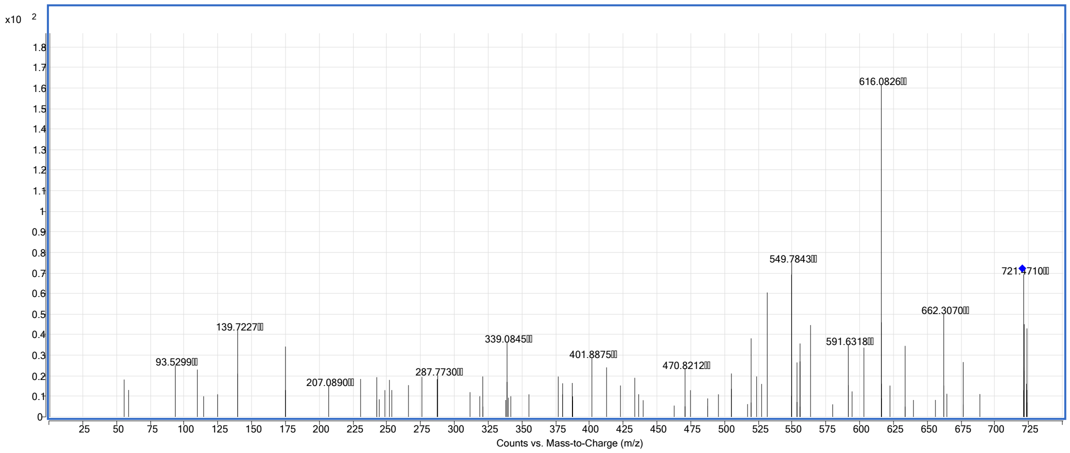

Supplement: Supplementary file 1 [file DataSheet_1.pdf]
